# Bacteriocin AS-48 and High Hydrostatic Pressure as Hurdles in a Vegetable Cream upon Temperature Abuse

**DOI:** 10.3390/microorganisms14040892

**Published:** 2026-04-16

**Authors:** Javier Rodríguez López, Rosario Lucas López, Mᵃ José Grande Burgos, Antonio Gálvez, Rubén Pérez Pulido

**Affiliations:** Microbiology Division, Department of Health Sciences, Faculty of Experimental Sciences, University of Jaén, 23071 Jaén, Spain; rlucas@ujaen.es (R.L.L.); mjgrande@ujaen.es (M.J.G.B.); agalvez@ujaen.es (A.G.); rppulido@ujaen.es (R.P.P.)

**Keywords:** vegetable cream, bacteriocin, high hydrostatic pressure, biodiversity

## Abstract

Refrigerated, ready-to-eat (RTE) vegetable foods are widely consumed. Microorganisms may proliferate in these foods during cold chain breaks, increasing the risks for food spoilage and foodborne disease. Despite the increasing use of novel non-thermal preservation technologies, the comprehensive impact of these hurdles on the broad taxonomic structural dynamics of the food microbiota during temperature abuse remains poorly understood. To address this gap, we determined the impact of bacteriocin AS-48 and a high hydrostatic pressure (HHP) treatment, both individually and in combination, on the microbial load and bacterial diversity of a refrigerated vegetable cream upon temperature abuse. Counts of aerobic mesophilic bacteria (37 °C, 24 h) increased significantly (*p* < 0.05) in controls during temperature abuse, but not in samples treated with bacteriocin, HHP or both. Amplicon-sequencing analysis indicated that the initial microbiota of control samples was composed mainly of *Pseudomonadota* (74.50%), followed by *Bacillota* (21.19%) and *Actinobacteriota* (3.69%). *Bacillota* became the predominant group during refrigerated storage (87.21 to 99.48%). After temperature abuse, control samples had lower relative abundances of *Bacillota* during storage and higher relative abundances of *Pseudomonadota*, *Bacteroidota* and *Actinobacteriota*. All treated samples (irrespective of the treatment) showed lower relative abundances of *Bacillota* during storage compared to untreated controls without temperature abuse. Genus *Bacillus* was the predominant group in the control samples during storage. *Acinetobacter* was associated with temperature abuse. In conclusion, both enterocin AS-48 and HHP can be effective hurdles, not only by preventing bacterial proliferation but also by influencing the dynamics of the microbial community associated with spoilage in vegetable creams exposed to inappropriate temperature conditions.

## 1. Introduction

Food microbiology plays a crucial role in food safety, as many processed products are subject to microbial challenges that can compromise their quality, safety and shelf life [1]. Vegetable creams, which have gained popularity as healthy and convenient foods [2], present a favorable environment for microbial growth due to their nutritional composition, water activity and moderate pH [3]. Furthermore, specific vegetable components, such as polysaccharides, can significantly modulate microbial community structure and their metabolic fermentation profiles [4]. The prevalence of the *Bacillus cereus* group in ready-to-eat (RTE) food products, such as vegetable spreads, represents a rising food safety concern worldwide. The widespread distribution and public health threat posed by these opportunistic pathogens in the retail sector have been consistently highlighted worldwide and across different continents, as demonstrated by recent studies conducted in Europe [5] and Asia [6]. Therefore, ensuring the safety and microbiological stability of these products is essential for their market acceptance and to prevent public health risks, as was clearly reflected in the World Health Organization (WHO) strategic planning meeting on food safety [7].

Emerging strategies to control microbial growth in minimally processed foods include the use of bacteriocins and non-thermal processing technologies such as high hydrostatic pressure (HHP) [8,9,10]. Bacteriocin AS-48, produced by *Enterococcus faecalis*, is a class IIc cyclic enterocin which has bactericidal activity against a large number of Gram-positive bacteria of importance in food, such as *Listeria*, *Bacillus* and *Clostridium* sp. [11], making it an effective tool for microbial control in food [12,13]. On the other hand, HHP is a technology widely used in the food industry to inactivate pathogenic and spoilage microorganisms without significantly altering the organoleptic and nutritional characteristics of the products [14,15,16,17].

Although both bacteriocins and HHP have been shown to be effective independently, recent studies suggest that the combination of both technologies could offer a synergistic effect [18], increasing the reduction in microbial load and reducing the emergence of resistant microorganisms and food-relevant microorganisms such as *Listeria monocytogenes* [19]. However, the effects of these combined strategies on the microbiota of specific foods, especially under conditions of temperature abuse, have not yet been widely explored.

Refrigerated, ready-to-eat vegetable foods are widely consumed. However, microorganisms surviving mild thermal processing and those arriving at the food during post-process contamination may proliferate in the food during storage if the cold chain breaks, raising the risks for food spoilage and foodborne disease. The aim of the present study was to determine the impact of bacteriocin AS-48 and HHP treatment, both individually and in combination, on the microbial load and bacterial diversity of a refrigerated-stored, ready-to-eat commercial vegetable cream composed mainly of pumpkin and carrot in order to improve the microbial stability of this food product under temperature abuse conditions. We also applied a culture-independent approach based on amplicon sequencing in order to gain insights into the microbiota of the vegetable cream. The findings of this study provide relevant information on the efficacy of these preservation strategies in a model food, providing new perspectives for the development of more efficient and safer preservation systems.

## 2. Materials and Methods

### 2.1. Preparation of Puree Samples

The refrigerated, ready-to-eat carrot and pumpkin puree was purchased in a well-known supermarket in Jaén (Spain) just before processing. The selection criteria of the product included being a minimally processed (refrigerated) food and free of added chemical preservatives (such as sorbic acid or benzoic acid), to avoid interfering with the tests. Only products with intact packaging and whose expiration date fell within the first third of their shelf life at the time of purchase were included. The puree was distributed in polyethylene-polyamide bags (preparing a total of 96 bags, 10 g each) under refrigeration to allow two replicates per sampling point and treatment. At time 0, samples (*n* = 24 per treatment) were treated by high-hydrostatic pressure, supplemented with enterocin AS-48 or treated with enterocin AS-48 plus high hydrostatic pressure. Control samples received no treatment. All the samples were refrigerated and stored at 4 °C for up to 30 days. On day 2, half of the samples (including controls and treatments) were incubated for 24 h at room temperature (simulating a temperature abuse event) and then refrigerated again. The specific implementation of the temperature abuse simulation at early storage (day 2) was strategically selected to represent a worst-case scenario. At specific times during incubation (days 0, 2, 3, 7, 15, 30), duplicate samples were drawn for each treatment and prepared for the culture-dependent and culture-independent microbiological analyses as described below.

### 2.2. Enterocin AS-48 Treatment

Enterocin AS-48 was prepared as described elsewhere [20]. Briefly, the bacteriocin producer strain was cultured in a complex medium containing 0.2% Casaminoacids (Difco, Detroit, MI, USA), 0.2% brain heart infusion (BBL, Cockeysville, MD, USA), 1% glucose, 0.1% yeast nitrogen base (YNB; Difco, Detroit, MI, USA), and 0.001% MgSO_4_·7H_2_O (PanReac, Barcelona, Spain) dissolved in 0.1 M sodium phosphate buffer (pH 7.2). After separation of bacterial cells by centrifugation, the bacteriocin was recovered from the cultured broths by cation exchange chromatography followed by dialysis through benzoylated cellulose tubing (MW cutoff, 2000; Sigma, St. Louis, MO, USA) against sterile saline solution. The bacteriocin preparation was cleaned through low protein-binding filters (0.22 μm pore size, Millex GV; Millipore Corp., Belford, MA, USA) under sterile conditions before use. Samples were supplemented with enterocin AS-48 to a final concentration of 50 µg/g (or 7.5% vol/vol of an equivalent volume of sterile saline solution without bacteriocin for control and HHP-treated samples), mixed well and stored refrigerated at 4 °C for up to 30 days. The biological activity of the purified enterocin AS-48 prepared under this standardized protocol corresponds to a known specific activity of 1700 AU/mg against the sensitive indicator strain *Enterococcus faecalis* S-47 [20]. The final chosen treatment dosage of 50 µg/g was established based on previous investigations demonstrating optimal antimicrobial efficacy against foodborne microbiota without compromising the food matrix. Furthermore, regarding its stability, enterocin AS-48 inherently maintains its targeted antimicrobial activity effectively during extended refrigerated storage at 4 °C in challenging conditions.

### 2.3. High-Hydrostatic Pressure Treatments

High-hydrostatic pressure (HHP) treatments were carried out by using a Stansted Fluid Power LTD HP equipment (SFP, Harlow, UK), equipped with a 2.5 L vessel capable of operating in a pressure range of 0 to 700 MPa. The system was equipped with an electrical heating unit (SFP) operating in a temperature range from room temperature up to 90 °C. The following HHP treatments were applied for 8 min: 600 MPa at 55 °C. These specific parameters were selected based on their industrial relevance for High-Pressure Thermal Processing (HPTP). While 600 MPa represents the maximum standard limit for commercial cold pasteurization, standard room-temperature HHP often fails to inactivate highly resistant bacterial spores or pressure-tolerant enzymes. Therefore, the application of moderately elevated temperature (55 °C) was specifically implemented to achieve a synergistic lethal effect against the resistant bacterial background, without causing thermal degradation of the matrix. Come-up speed was 75 MPa/min. Decompression was almost immediate. Pressurization fluid was water with added 10% propylenglycol (Panreac, Madrid, Spain). Control and bacteriocin-treated samples (without HHP treatment) were run in parallel. The combined treatment (bacteriocin and HHP) was carried out sequentially: first, enterocin AS-48 was added, and then the HHP treatment was performed. Furthermore, the circular structure of enterocin AS-48 confers it with exceptional stability, allowing it to retain its activity under high-pressure thermal treatment (HPTP) conditions, as has been demonstrated previously [13]. Right after treatments, all samples were placed on an ice basket for 30 min and then stored at 4 °C for up to 30 days.

### 2.4. Microbiological Analysis and pH Measurement

Puree samples (1 mL) were serially diluted in sterile saline solution (9 mL) with vortexing. The undiluted puree and the serial dilutions obtained were spread-plated (0.1 mL per plate) in triplicate on the nutrient-rich medium, trypticase soya agar (TSA; Scharlab, Barcelona, Spain). The plates were incubated at 37 °C instead of 30 °C for 24 h for the determination of aerobic mesophiles to specifically target and quantify microbial populations capable of growing at human physiological temperatures, which are of primary interest for food safety/clinical relevance. The mean viable cell count was expressed as log_10_ colony-forming units (CFU) per gram of sample. The limit of detection (LOD) for the microbiological counts was 10 CFU/g (corresponding to 1 log CFU/g). For statistical analysis, counts below the detection limit were assigned a value of the LOD itself. The pH of the puree samples was determined with a pH meter (Crison Instruments, S.A., Barcelona, Spain).

### 2.5. DNA Extraction

Aliquots (5 mL) of each sample replicate were mixed with 10 mL sterile saline solution and homogenized in a Stomacher 400 (Seward, Worthing, UK) for 1 min. Homogenates were centrifuged at 600× *g* for 5 min to remove solids. Aliquots (1.5 mL) of the resulting supernatants were transferred to an Eppendorf test tube and centrifuged at 13,500× *g* for 5 min to recover microbial cells. The pellets were resuspended in 0.5 mL of sterile saline each. Then, propidium monoazide (PMA, GenIUL, S.L, Barcelona, Spain) was added to a final concentration of 50 µM. Samples were incubated in the dark for 10 min, followed by light exposure for 15 min using an LED photo-activation system (Photo-Activation System, GenIUL) to cross-link the dye to the DNA. This step was performed to block subsequent PCR amplification of DNA from dead cells [21,22]. DNA from PMA-treated cells was extracted using a DNeasy PowerSoil Kit (Qiagen, Madrid, Spain). The resulting DNA from the two replicates from each bag and from the same sampling point was pooled into a single sample. The quality and quantity of the extracted DNA were determined by QuantiFluor^®^ ONE dsDNA system (Promega, Madison, WI, USA).

### 2.6. DNA Sequencing and Analysis

The 16S rDNA V3-V4 regions were amplified targeting the V3 and V4 regions following Illumina Metagenomic Sequencing Library Preparation protocol (Illumina, Inc., San Diego, CA, USA). The following primers (including Illumina adapters) were used: Forward: 5′-TCGTCGGCAGCGTCAGATGTGTATAAGAGACAGCCTACGGGNGGCWGCAG-3′ and Reverse: 5′-GTCTCGTGGGCTCGGAGATGTGTATAAGAGACAGGACTACHVGGGTATCTAATCC-3′ [23]. Microbial genomic DNA (5 ng/ μL in 10 mM Tris pH 8.5) was used to initiate the protocol. After 16S rDNA gene amplification, the multiplexing step was performed using Nextera XT Index Kit (Illumina). A total of 1 μL of the PCR product was run on a Bioanalyzer DNA 1000 chip to verify the size (expected size ~550 bp). To ensure the absence of environmental or reagent contamination, negative controls were included for both the DNA extraction process (blank samples) and the PCR amplification (no-template controls). No positive/mock community was used in this study.

After size verification, the libraries were sequenced using a 2 × 300 pb paired-end run on a MiSeq Sequencer according to the manufacturer’s instructions (Illumina). Quality control of the sequencing run showed a high performance, yielding an average of 121,928 raw reads per sample, with >99.3% of the initial sequences successfully passing the preliminary quality filters. Sequencing data were processed using nf-core/ampliseq version 2.13.0 [24] of the nf-core collection of workflows [25], utilizing reproducible software environments from the Bioconda [26] and Biocontainers [27] projects. Data quality was evaluated with FastQC (v0.12.1) [28] and summarized with MultiQC (v1.27) [29]. Sequences were processed sample-wise (independent) with DADA2 (v1.30.0) [30] to eliminate PhiX contamination, discard reads with >2 expected errors, correct errors, and remove PCR chimeras. Specifically, the key DADA2 filtering parameters included a forward sequence truncation length (truncLen) of 240 bp and a reverse sequence truncation length of 200 bp, with a maximum expected error (maxEE) threshold set to 2 for both reads. PCR chimeras were identified and removed using the default consensus method in DADA2. Taxonomic classification was performed by DADA2 leveraging the reference database ‘Silva 138.2 prokaryotic SSU’ [31]. Overall, final read retention following DADA2 denoising and merging ranged strictly between 71.7% and 99.9% across samples.

Contaminant Amplicon Sequence Variants (ASVs) were identified and removed by filtering sequences prevalent in the negative controls using manual filtering based on prevalence. Additionally, strict taxonomic filtering was applied to all samples by systematically discarding any ASVs assigned to non-target background DNA, such as mitochondria and chloroplasts. Read counts before and after the pipeline quality processing and taxonomic filtering steps are detailed per sample in Supplementary Table S1. Regarding rare taxa, the pipeline maintained all detected high-quality sequences without setting a strict sequence-loss filter. Instead, during downstream statistical analysis and graphical representation in R, any ASV representing less than 3% of the relative abundance within a sample was dynamically agglomerated into a unified “rest < 3%” category. This approach prevented the loss of read depth for cumulative assessments while facilitating the interpretation of the core taxonomic composition. The final ASV sequences, abundance tables, and DADA2 taxonomic assignments were loaded into QIIME2 (v2024.10) [32]. Subsequent analyses, conducted through QIIME2, included assessments of alpha and beta diversity as well as interactive visualizations of the microbial taxonomic composition. Final outputs were structured into R-compatible objects for further statistical analysis and custom data exploration.

### 2.7. Statistical Analysis

A total of 96 samples were analyzed, corresponding to the four treatments (Control, bacteriocin, HHP, and combination), two defined storage conditions (strict refrigeration vs. temperature abuse), and the six different sampling times. The experimental design consisted of two independent biological replicates (i.e., two independent production batches of vegetable cream). For each sample, serial dilutions were plated in technical triplicate, and mean values were calculated before further analysis. Bacterial counts were log-transformed prior to statistical analysis to meet the assumptions of normality and homogeneity of variances. Culture-dependent microbiological data were analyzed by one-way analysis of variance (ANOVA), performed independently for each sampling time and treatment. When significant differences were detected, means were compared using Tukey’s post hoc test. Mean values and standard deviations were calculated using Microsoft Excel (Microsoft Corp., Redmond, WA, USA). Bacterial community diversity was evaluated by Principal Coordinates Analysis (PCoA) based on Bray–Curtis distance matrices using the scikit-bio library in Python (v3.9.1). Differences were considered statistically significant at *p* < 0.05.

## 3. Results

### 3.1. Effect of Different Treatments and Storage Time on Microbial Loads

Bacterial counts in control samples not subjected to temperature abuse (C) remained stable throughout storage, with values between 2.54 and 2.25 log CFU/g (Table 1). In contrast, viable cell counts from control samples subjected to temperature abuse (CAB) increased significantly (*p* < 0.05) from day 3 until the end of storage, reaching values between 6.47 and 7.73 log CFU/g.

The bacteriocin-treated samples (B) showed significantly lower viable counts (*p* < 0.05) on days 3, 7 and 30 compared to control samples and from day 3 on compared to the temperature abuse samples (CAB). Interestingly, viable counts did not increase in the bacteriocin-treated samples after temperature abuse (BAB), and decreased below detectable levels on days 15 and 30.

Treatments with HHP (H) and bacteriocin in conjunction with HHP (BH) reduced viable counts below the detection limit (<1.0 log CFU/g) for the whole storage period. These treatments also reduced viable counts below detection limits in the samples with temperature abuse (HAB, BHAB).

The pH of samples with temperature abuse (CAB) decreased significantly (*p* < 0.05) at days 7 to 30 (Table 1). By contrast, the pH of control samples was more stable or increased significantly (*p* < 0.05) at the end of storage (T30). Many of the treated samples (except H treatment) also showed significantly higher (*p* < 0.05) pH values at the end of storage (T15, T30 or both).

### 3.2. Bacterial Diversity

Most of the treated samples did not yield bacterial DNA of sufficient quantity/quality for amplicon sequencing (possibly because of the low numbers of viable cells and the interference of PMA with dead cells and cells with a compromised cytoplasmic membrane resulting from HHP or bacteriocin action), and therefore, they were excluded from the bacterial diversity analysis (see Supplementary Table S2). The number of reads assigned to Amplicon Sequence Variants (ASVs) after taxonomic classification and filtering of unwanted taxa and alpha biodiversity indices (Shannon, Simpson and Chao) are shown in Table 2. The number of reads ranged from 56,674 to 166,157. All sequenced samples show similar Shannon, Simpson and Chao-1 indices, except the untreated control samples not subjected to temperature abuse, whose values are lower from day 3 to the end of storage. In addition, it is worth mentioning the sample CAB3 with a value of the Chao-1 biodiversity index higher than the rest of the samples.

**Figure 1:**
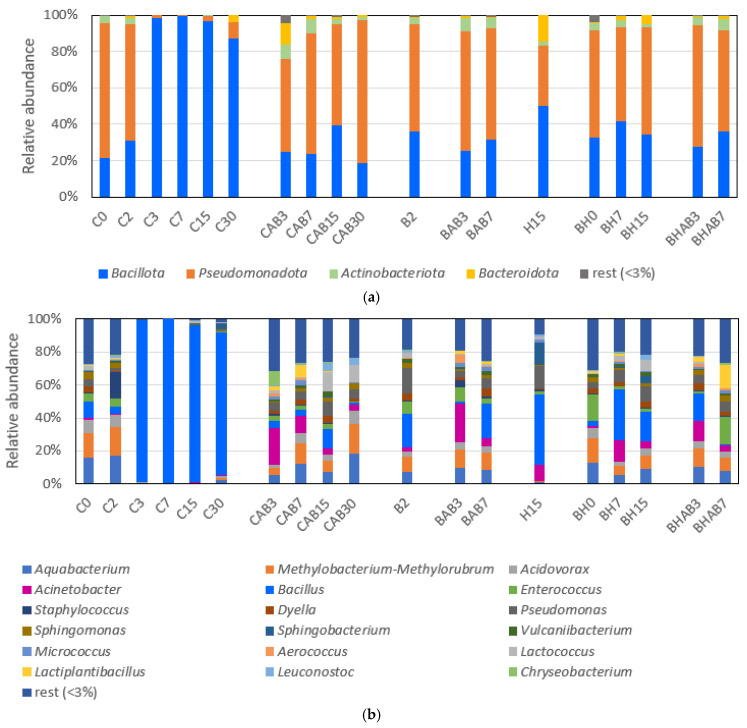
Bacterial diversity of samples at the phylum (**a**) and genus level (**b**). Untreated control (C), bacteriocin-treated (B), HHP-treated (H), and bacteriocin- and HHP-treated (BH). Samples with abuse temperature are indicated with “AB”. Each number corresponds to the different sampling times (in days) during storage.

Taxonomic analysis indicated that *Pseudomonadota* and *Bacillota* were the two main phyla represented in the samples, followed by *Actinobacteriota* and *Bacteoidota* (Figure 1a). During the first 2 days of refrigerated storage, *Pseudomonadota* was the most abundant phylum, followed by *Bacillota* (Figure 1a). The *Pseudomonadota* group was mainly represented by members of the genera *Aquabacterium*, *Methylobacterium-Methylorubrum* and *Acidovorax* (Figure 1b). From day 3 until the end of the storage, *Bacillota* (represented mainly by genus *Bacillus*) became the predominant ASV, reaching relative abundances above 99%. By contrast, the control samples subjected to abusive temperature (CAB) showed high relative abundances of *Pseudomonadota* along with an increase in *Actinobacteriota* and *Bacteroidota*. *Acinetobacter*, *Aquabacterium*, *Methylobacterium-Methylorubrum*, *Pseudomonas* and *Chryseobacterium* were the main representative genera detected in CAB samples.

The few samples treated with bacteriocin or bacteriocin plus high hydrostatic pressure that could be analyzed showed taxonomic profiles at the phylum level resembling the control samples at early storage, with *Pseudomonadota* being the most abundant phylum, followed by *Bacillota*. The most abundant genera were *Pseudomonas*, *Sphingomonas*, *Methylobacterium-Methylorubrum, Aquabacterium, Acinetobacter, Enterococcus* or *Bacillus*.

**Figure 2:**
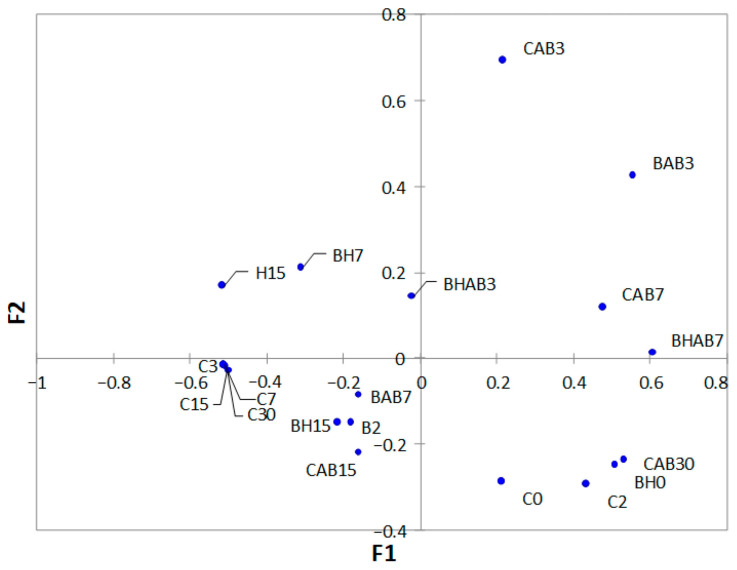
Principal Coordinates Analysis (PCoA) based on Bray–Curtis distances showing the beta diversity of untreated control (C), bacteriocin-treated (B), HHP-treated (H) and bacteriocin and HHP-treated (BH) samples. Samples with abuse temperature are indicated with “AB”. Each number corresponds to the different sampling times (in days) during storage.

Principal coordinate analysis (PCoA) was performed with control samples and those treated samples with good DNA quantity/quality for amplicon sequencing (Figure 2). A subgroup formed by the control samples taken from day 3 of storage and not subjected to abusive temperature (C3, C5, C7 and C30) can be observed, which are clearly distant from the rest of the samples. Furthermore, the storage times showed in some cases an influence on the bacterial composition regardless of the type of treatment to which they were subjected, as in the case of the subgroups formed by the samples taken at the beginning of storage (C0, C2 and BH0).

## 4. Discussion

The results obtained in the present study indicated that the concentrations of viable cell count (aerobic mesophiles at 37 °C, 24 h) in the vegetable puree remained low during storage. However, viable cell counts rapidly increased remarkably with a 24 h temperature abuse period, compromising the microbiological quality and safety of the product. The negative effect on the microbiological safety of food when experiencing a break in the cold chain has been demonstrated by previous studies. Reference [33] reported similar results in a study with courgette puree. An increase in temperature during storage (from 4 °C to room temperature) led to an increase in aerobic mesophiles counts from 3.9 to 7.8 log CFU/g. Similar results regarding high levels of contamination of vegetable foods were reported by [34], while other studies reported lower contamination levels [35].

Previous studies already reported the high efficacy of HHP treatments on vegetables such as pumpkin [36]. Our study has shown that the use of HHP treatment at 55 °C (alone or in combination with bacteriocin) prevents bacterial proliferation during temperature abuse conditions. This would be expected considering the low initial microbial load of the puree samples and the enhanced antimicrobial activity obtained when HHP treatments are applied in combination with moderate heat [9,37,38,39]. Importantly, the combination of HHP treatments with heat has also been reported to improve inactivation of bacterial endospores [40,41,42], which may represent one of the main risks in ready-to-eat, slightly processed vegetable foods. According to the results obtained in the present study, the HHP treatment could be recommended in order to diminish the risks of microbial proliferation in the vegetable cream upon exposure to temperature abuse conditions.

Although not as effective as HHP, the addition of AS-48 could also be considered as a barrier against bacterial proliferation in the puree samples during temperature abuse conditions. The effectiveness of enterocin AS-48 as a biopreservative has already been tested in ready-to-eat vegetable foods against aerobic mesophilic endospore-forming bacteria by [43] and in several types of vegetable foods [12,13,44]. Similar to nisin [41], enterocin AS-48 improves microbial inactivation and reduces the recovery of survivors when tested in combination with HHP treatments [45]. HHP causes multiple cell damages, including protein denaturation and destabilization of the structural and functional integrity of bacterial membranes [16], while enterocin AS-48 permeabilizes the bacterial cytoplasmic membrane, leading to energy depletion (and in some cases to cell autolysis) [13]. We suggest that HHP-induced destabilization of the Gram-negative outer membrane facilitates bacteriocin entry and cell death, while bacteriocin-mediated pore formation and energy depletion prevent the repair of HHP-induced sublethal injuries. However, a synergistic effect of AS-48 and HHP could not be demonstrated in the carrot and pumpkin puree, likely due to the very low initial microbial load concentrations.

The analysis of bacterial diversity indicated a high relative abundance of *Pseudomonadota* at the beginning of storage, with a progressive compositional shift in refrigeration conditions without temperature abuse towards a greater relative abundance of *Bacillota*, with *Bacillus* appearing as the clearly dominant genus. We hypothesize that the observed changes could be associated with the selective proliferation of psychrotrophic *Bacillus* strains, proportionally outcompeting other members of the bacterial community that may be present in the samples. However, under conditions of temperature abuse, the microbiota was characterized by a persistence of *Pseudomonadota* and the increase in relative abundance of *Acinetobacter* (among others). This persistence could be linked to the inherent ability of certain *Pseudomonadota*—specifically *Pseudomonas*—to develop complex spatial patterns and cell-to-cell communication that facilitate environmental resilience and biofilm formation [46]. In contrast, the rest of the treated samples (irrespective of treatment) were generally linked to a microbiological profile dominated by the phylum *Pseudomonadota*, correlating with a reduced relative detection of *Bacillota* and less development of undesirable genera such as *Bacillus* throughout storage. It is important to note a limitation regarding the analysis of bacterial diversity in the treated samples. Given that the treatments (particularly HHP and its combinations) were highly effective at reducing the microbial load and that PMA was used to remove DNA from dead cells, many treated samples did not yield sufficient DNA for amplicon sequencing. Consequently, the diversity profiles obtained for the treated samples are biased towards the small subpopulation of surviving bacteria. Therefore, claims regarding the taxonomic composition of the treated samples should be interpreted with caution.

The results of the bacterial diversity analysis should be of concern, considering the risks of spread of antibiotic-resistant bacteria [47] and outbreaks caused by pathogens present in vegetables, such as the recent case reported by [48], in which a virulent strain of the genus *Bacillus* associated with vegetable food was isolated from a foodborne outbreak and caused three deaths. Being a soil bacterium, *Bacillus* can spread easily to vegetable foods. A previous study reported a prevalence of 20% for *Bacillus cereus* in cooked chilled foods containing vegetables, and most of the *B. cereus* strains isolated were psychrotrophic [49]. Most worrying, members of *Bacillus* can produce different toxins [50,51], among which cereulide may cause multi-organ failure [52]. Regarding the increase in relative abundance of *Acinetobacter* in the vegetable cream during temperature abuse, previous studies have reported the presence of this bacterium from drinking water and foods [53], including vegetables [54]. *Acinetobacter* has been reported in carrot wash water [55], and multidrug-resistant (MDR) *A. baumannii* and *A. lwoffii* have been detected in carrots originating from crops irrigated with water from the Jakara River in Nigeria, into which domestic, hospital, commercial, and industrial sewage is discharged [56]. While most *Acinetobacter* spp. are non-pathogenic environmental organisms, those species adapted to clinical environments are now causing serious health problems [57]. Further studies are needed that include the isolation and species-specific genomic identification of potential *Bacillus* and *Acinetobacter* strains present in purees, as well as the characterization of their potential to produce toxins and spread antibiotic resistance genes. It would also be of interest to conduct future studies focused on determining the inactivation rates of the isolated spores to understand the persistence dynamics of *Bacillus* species in these treated purees.

In conclusion, the results obtained in the present study indicate that both enterocin AS-48 and HHP can be effective hurdles that significantly reduce bacterial proliferation in the vegetable cream, even when the product is exposed to unsuitable temperature conditions.

## Figures and Tables

**Table 1:** Viable cell counts (aerobic mesophiles at 37 °C, 24 h) and pH of samples, subjected or not to temperature abuse.

Viable Counts	T0	T2	T3	T7	T15	T30
C	2.54 ± 0.16	2.29 ± 0.05	2.16 ± 0.11	2.99 ± 0.11	1.91 ± 0.29	2.25 ± 0.1
CAB	2.54 ± 0.07	2.06 ± 0.02	6.47 ± 0.01 ^a^	7.73 ± 0.17 ^a^	7.59 ± 0.06 ^a^	6.96 ± 0.13 ^a^
B	1.65 ± 0.06	1.93 ± 0.04	1.65 ± 0.07 ^b,c^	1.63 ± 0.21 ^b,c^	1.63 ± 0.21 ^c^	1.74 ± 0.06 ^b,c^
BAB	1.72 ± 0.17	1.72 ± 0.33	1.54 ± 0.08 ^b,c^	1.23 ± 0.11 ^b,c^	<1	<1
H	<1	<1	<1	<1	<1	<1
HAB	1	<1	<1	<1	<1	<1
BH	<1	<1	<1	<1	<1	<1
BHAB	<1	<1	<1	<1	<1	<1
**pH**	**T0**	**T2**	**T3**	**T7**	**T15**	**T30**
C	5.49 ± 0.01 ^a^	5.54 ± 0.06 ^a^	5.56 ± 0.06 ^a^	5.69 ± 0.08 ^a,b^	5.58 ± 0.04 ^a^	5.96 ± 0.08 ^b^
CAB	5.50 ± 0.01 ^a^	5.56 ± 0.03 ^a^	5.48 ± 0.07 ^a,c^	5.42 ± 0.03 ^c^	5.34 ± 0.06 ^c^	5.47 ± 0.07 ^a,c^
B	5.50 ± 0.03 ^a^	5.54 ± 0.04 ^a^	5.52 ± 0.06 ^a^	5.46 ± 0.08 ^a^	5.81 ± 0.06 ^b^	5.93 ± 0.07 ^b^
BAB	5.51 ± 0.01 ^a^	5.53 ± 0.04 ^a^	5.54 ± 0.01 ^a^	5.52 ± 0.03 ^a^	5.97 ± 0.10 ^b^	5.88 ± 0.08 ^b^
H	5.60 ± 0.03 ^a^	5.62 ± 0.03 ^a^	5.64 ± 0.04 ^a^	5.71 ± 0.07 ^a^	5.42 ± 0.11 ^a^	5.63 ± 0.10 ^a^
HAB	5.59 ± 0.01 ^a^	5.61 ± 0.01 ^a^	5.60 ± 0.05 ^a,d^	5.45 ± 0.07 ^a^	5.81 ± 0.04 ^b,d^	5.95 ± 0.07 ^b^
BH	5.60 ± 0.03 ^a^	5.59 ± 0.04 ^a^	5.61 ± 0.04 ^a^	5.36 ± 0.11 ^a,c^	6.07 ± 0.12 ^b^	5.82 ± 0.07 ^a,b^
BHAB	5.58 ± 0.03 ^a^	5.60 ± 0.03 ^a^	5.57 ± 0.12 ^a^	5.48 ± 0.08 ^a^	5.96 ± 0.10 ^b^	5.65 ± 0.12 ^a^

Data are expressed as the mean ± standard deviation (SD) of 3 independent biological replicates (*n* = 3). Samples include untreated control (C), bacteriocin-treated (B), HHP-treated (H), and bacteriocin- and HHP-treated (BH) groups. Samples with temperature abuse are indicated with “AB”. The storage time in days is indicated by “T”. Statistical significance (*p* < 0.05) of viable counts: a, significantly higher than untreated control (with temperature abuse or not) counts obtained at time 0 and 2, and significantly higher than untreated controls without temperature abuse at the same storage time; b, significantly lower than untreated controls without temperature abuse at the same storage time; c, significantly lower than untreated controls subjected to temperature abuse at the same storage time. In the case of pH values, different superscript letters (a, b, c, d) within the same row indicate significant differences (*p* < 0.05) among storage times for each treatment.

**Table 2:** Number of reads and alpha diversity indices at genus level of untreated control (C), bacteriocin treated (B), HHP treated (H) and bacteriocin and HHP treated (BH) samples.

Sample	No. Reads	Shannon	Simpson	Chao 1
Untreated controls				
C0	113,163	0.94	3.77	238
C2	96,211	0.91	3.37	177
C3	101,240	0.20	0.54	49
C7	166,157	0.21	0.51	57
C15	90,838	0.25	0.77	107
C30	97,888	0.39	1.22	153
CAB3	105,302	0.97	4.63	492
CAB7	105,920	0.95	4.03	270
CAB15	81,585	0.96	4.12	319
CAB30	79,818	0.92	3.62	231
Bacteriocin				
B2	65,732	0.93	3.62	262
BAB3	93,398	0.93	3.62	215
BAB7	87,754	0.94	3.88	307
High hydrostatic pressure				
H15	71,670	0.79	2.53	224
Bacteriocin + High hydrostatic pressure				
BH0	96,752	0.94	3.91	285
BH7	76,316	0.89	3.55	335
BH15	56,674	0.94	3.78	227
BHAB3	90,103	0.94	3.84	247
BHAB7	89,248	0.95	3.93	315

Samples: untreated control (C), bacteriocin treated (B), HHP treated (H) and bacteriocin and HHP treated (BH). Samples with abuse temperature are indicated with “AB”. Each number corresponds to the different sampling times (in days) during storage. The Shannon and Simpson indices are estimators of both species richness and evenness, with more emphasis on richness (Shannon) or evenness (Simpson). Only the treated samples with good DNA quantity/quality for amplicon sequencing are included.

## Data Availability

The original contributions presented in this study are included in the article/Supplementary Material. Further inquiries can be directed to the corresponding author.

## Supplementary Materials

The following supporting information can be downloaded at: https://www.mdpi.com/article/10.3390/microorganisms14040892/s1, Table S1. Sequencing Read Counts and Retention Rates. Table S2. List of samples excluded from the amplicon-sequencing diversity analysis.
