# Supplementary material for: Bacteriocin AS-48 and High Hydrostatic Pressure as Hurdles in a Vegetable Cream upon Temperature Abuse
Source: Microorganisms. 2026 Apr 16;14(4):892. doi: 10.3390/microorganisms14040892 (PMC13118743; doi:10.3390/microorganisms14040892)
Supplement: Supplementary file 1 [file microorganisms-14-00892-s001.zip › microorganisms-4234172-supplementary.pdf]

Supplementary Table S1. Sequencing Read Counts and Retention Rates.

Sample ID	Treatment Group	Raw Reads (Before Processing)	Final Retained Reads (After Processing)	Retention (%)
C0	control	146,818	113,163	77.08%
BH0	bacteriocin+HHP	123,716	96,752	78.20%
C2	control	128,231	96,211	75.03%
B2	bacteriocin	85,829	65,732	76.58%
C3	control	120,399	101,24	84.09%
CAB3	control	143,466	105,302	73.40%
BAB3	bacteriocin	122,936	93,398	75.97%
BHAB3	bacteriocin+HHP	114,126	90,103	78.95%
C7	control	199,51	166,157	83.28%
BH7	bacteriocin+HHP	112,473	76,316	67.85%
CAB7	control	140,77	105,92	75.24%
BAB7	bacteriocin	115,038	87,754	76.28%
BHAB7	HHP	124,513	80,92	64.99%
C15	control	114,53	90,838	79.31%
H15	HHP	98,538	71,67	72.73%
BH15	bacteriocin+HHP	78,123	56,674	72.54%
CAB15	control	127,695	81,585	63.89%
C30	control	121,939	97,888	80.28%
CAB30	control	119,562	79,818	66.76%

Samples: untreated control (C), bacteriocin treated (B), HHP treated (H) and bacteriocin and HHP treated (BH). Samples with abuse temperature are indicated with "AB". Each number corresponds to the different sampling times (in days) during storage.

Supplementary Table S2. List of samples excluded from the amplicon-sequencing diversity analysis.

Group treatment	Excluded Sampling Times (Days)	Reason for Exclusion
Negative control	All sampling points	Low DNA quantity/quality
Bacteriocin (B)	0, 3, 7, 15, 30	Low DNA quantity/quality
Bacteriocin + Temp. Abuse (BAB)	15, 30	Low DNA quantity/quality
High Hydrostatic Pressure (H)	0, 2, 3, 7, 30	Low DNA quantity/quality
HHP + Temp. Abuse (HAB)	0, 2, 3, 7, 15, 30	Low DNA quantity/quality
Bacteriocin + HHP (BH)	2, 3, 30	Low DNA quantity/quality
Bacteriocin + HHP + Temp. Abuse (BHAB)	15, 30	Low DNA quantity/quality

(Note: All Control samples (C) and Control samples with temperature abuse (CAB) yielded sufficient DNA and were included in the analysis).
